# Tissue miR-143 and miR-29b Expression as Prognostic Biomarkers in Patients with Resected Pancreatic Ductal Adenocarcinoma Receiving Adjuvant Chemotherapy

**DOI:** 10.3390/diagnostics16213473

**Published:** 2026-10-24

**Authors:** Dilruba Efe Keskin, Aysen Sagnak, Elif Erturk, Oguzhan Akgun, Nesrin Ugras, Gul Akin, Ahmet Bilgehan Sahin, Ferda Ari, Adem Deligonul

**Affiliations:** 1 Department of Internal Medicine, Faculty of Medicine, Bursa Uludag University, Bursa 16059, Turkey; dilrubaefeee@gmail.com; 2 Department of Biology, Faculty of Arts and Science, Bursa Uludag University, Bursa 16059, Turkey; aysensagnak43@gmail.com (A.S.); oguzhanakgun@uludag.edu.tr (O.A.); ferdaoz@uludag.edu.tr (F.A.); 3 Vocational School of Health Services, Bursa Uludag University, Bursa 16059, Turkey; eliferturk@uludag.edu.tr; 4 Department of Medical Pathology, Bursa Uludag University Hospital, Bursa 16059, Turkey; nesrinugras@uludag.edu.tr; 5 Department of Medical Oncology, School of Medicine, Bursa Uludag University, Bursa 16059, Turkey; gulakin@uludag.edu.tr (G.A.); absahin@uludag.edu.tr (A.B.S.)

**Keywords:** pancreatic ductal adenocarcinoma, microRNA, adjuvant chemotherapy, local recurrence, disease-free survival, prognosis

## Abstract

**Background/Objectives**: Pancreatic ductal adenocarcinoma (PDAC) carries one of the worst prognoses among solid tumors, and biomarkers that complement standard pathological variables in patients undergoing resection followed by adjuvant chemotherapy are still lacking. Here, we examined whether tumor expression of 10 selected microRNAs (miRNAs) is associated with recurrence patterns and survival in this clinical setting. **Methods**: Fifty patients with resected PDAC who received adjuvant chemotherapy at a single institution (2012–2022) were studied retrospectively. Expression of let-7c, miR-10b, miR-27a, miR-29b, miR-31, miR-34a, miR-142, miR-143, miR-17-5p, and miR-200c was measured by quantitative real-time PCR in formalin-fixed paraffin-embedded tumor tissue, with adjacent non-tumor pancreatic tissue available for 21 patients. Associations with local recurrence, metastasis, disease-free survival (DFS), and overall survival (OS) were assessed by logistic regression and Cox regression, with miRNA expression dichotomized at the median. **Results**: Seven miRNAs (let-7c, miR-27a, miR-29b, miR-31, miR-34a, miR-143, miR-200c) were downregulated in tumor tissue and miR-10b was upregulated (all *p* < 0.05). Higher tumor expression of miR-143 was associated with local recurrence, while higher miR-29b expression was associated with shorter disease-free survival. Adenosquamous histology was independently associated with shorter DFS and OS. **Conclusions**: Higher tumor expression of miR-143 and miR-29b was associated with local recurrence and shorter disease-free survival, respectively. These exploratory associations require validation in larger independent cohorts.

## 1. Introduction

Pancreatic ductal adenocarcinoma (PDAC) carries one of the worst prognoses among solid tumors, with five-year survival in the 2–9% range despite progress in surgical technique and systemic therapy [1,2]. While surgical resection followed by adjuvant chemotherapy is the only treatment with curative intent [3], most patients relapse within two years and the majority die of recurrent disease [4]. In this resected and chemotherapy-treated population, clinicians have few molecular tools to anticipate which patients will recur and how long they will survive [5,6]. Standard pathological variables, including margin status, lymph node involvement, perineural invasion, and histological subtype, account for only part of the observed prognostic heterogeneity [7], and validated biomarkers that may complement these features and refine post-operative risk stratification remain an unmet need [8].

MicroRNAs (miRNAs) are small non-coding RNA molecules of approximately 22 nucleotides that regulate gene expression post-transcriptionally by binding the 3′ untranslated region of target messenger RNAs and promoting their degradation or translational repression [9,10]. Depending on cellular context and on the spectrum of targets engaged, individual miRNAs behave as oncogenes or tumor suppressors [11,12]. Tissue miRNAs remain stable in formalin-fixed paraffin-embedded material and can be quantified reproducibly by RT-qPCR [13], a property that has favored their adoption as diagnostic and prognostic biomarkers in several solid malignancies [14]. In PDAC, distinct miRNA signatures distinguish tumor tissue from chronic pancreatitis and from normal pancreas [15], and several miRNAs participate in invasion, epithelial-to-mesenchymal transition, and chemoresistance in preclinical models [16,17].

Most miRNA studies in patients with PDAC have nevertheless been performed on heterogeneous cohorts that combine resectable, locally advanced, and metastatic disease [18], and the prognostic value of miRNAs has been studied less extensively in the specific setting of resected PDAC followed by adjuvant chemotherapy. Schultz and colleagues earlier proposed a five-miRNA prognostic index in tissue from operated patients, showing feasibility while also exposing the variability of single-cohort signatures [19]. More recent work has begun to address the gap directly. Yuan and colleagues reviewed circulating and tissue miRNAs as prognostic and predictive markers in PDAC, noting that tumor-tissue signatures specifically validated in adjuvantly treated cohorts remain scarce [20]. Mok and colleagues summarized the role of miRNAs in PDAC progression and metastasis, pointing to the small number of post-resection cohorts with adequate follow-up [21]. Huang and colleagues developed a serum miR-24 nomogram for prognosis in resected PDAC, showing the predictive potential of miRNA-based tools in this population while leaving open the question of which tumor-tissue miRNAs, measured at the time of resection, carry independent information beyond established pathological variables [22].

We therefore hypothesized that the abundance of a defined panel of tumor-tissue miRNAs would be associated with local recurrence, distant metastasis, disease-free survival (DFS), and overall survival (OS) in patients with resected PDAC who received adjuvant chemotherapy. The miRNAs were chosen to span complementary biological programs that have been implicated in PDAC and that are biologically plausible determinants of recurrence under cytotoxic pressure, namely epithelial-to-mesenchymal transition (EMT) (miR-200c [23], miR-10b [24]), stem-like and proliferative regulation (miR-34a [25], let-7c [26], miR-17-5p [27]), stromal and extracellular matrix biology (miR-29b [28], miR-143 [29]), invasion (miR-27a [30]), and immune or inflammatory regulation (miR-31 [31], miR-142 [32]). The aim of the present study was to examine whether the expression of these ten miRNAs in tumor tissue, compared with adjacent non-tumor tissue, correlates with recurrence patterns and survival in a single-center cohort of patients with resected PDAC who received adjuvant chemotherapy.

## 2. Materials and Methods

### 2.1. Study Design and Patients

This was a retrospective single-center study of consecutive patients with histologically confirmed PDAC who underwent surgical resection with curative intent and received adjuvant chemotherapy at the Department of Medical Oncology, Faculty of Medicine, Uludağ University (Bursa, Türkey), between January 2012 and December 2022. Patients were included if formalin-fixed paraffin-embedded (FFPE) tumor tissue of adequate quality was available and if they had completed at least one cycle of adjuvant chemotherapy. Fifty patients met these criteria. In 21 of them, paired FFPE blocks of histologically confirmed adjacent non-tumor pancreatic tissue were also available. Demographic data, histological subtype, tumor stage, perineural invasion, recurrence pattern, and survival data were retrieved from the medical records. The study was approved by the Bursa Uludağ University Clinical Research Ethics Committee (approval no. 2023-9/4) and was conducted in accordance with the Declaration of Helsinki.

### 2.2. Endpoints

The endpoints were local recurrence, distant metastasis, DFS, and OS. Local recurrence was defined as evidence of disease in the pancreatic bed or regional lymph nodes; distant metastasis as disease at extra-regional sites. DFS was calculated from surgery to first recurrence (local or distant) or death from any cause. OS was the interval from surgery to death from any cause. Patients alive and event-free at the last visit were censored at the date of last follow-up.

### 2.3. Selection of Candidate miRNAs

Candidate miRNAs were prioritized through a literature-based approach. A search of PubMed, Web of Science, Wiley Online Library, Taylor & Francis Online, and Elsevier ScienceDirect was performed through March 2023 with the queries “metastasis AND miRNA”, “pancreatic cancer AND miRNA”, and “metastatic pancreatic cancer AND miRNA”. Two text corpora were assembled from the retrieved records, and miRNA names were extracted with the Tokenizer version 0.3.0 and quanteda packages version 4.5.0 in R. The procedure returned 1270 miRNAs in the metastasis corpus and 1057 miRNAs in the pancreatic cancer corpus, with 145 miRNAs shared between the two sets. From this pool, ten miRNAs were retained on the basis of recurrence in the literature and complementary coverage of biological programs relevant to PDAC [21,22,23,24,25,26,27,28,29,30,31,32].

### 2.4. Tissue Handling and RNA Extraction

For each block, sections of 5–10 µm were cut and transferred to nuclease-free 1.5–2 mL microcentrifuge tubes. Deparaffinization was performed with 160 µL of Deparaffinization Solution (cat. no. 19093, Qiagen, Hilden, Germany), and total RNA was extracted with the miRNeasy FFPE Kit (Qiagen) according to the manufacturer’s instructions. RNA concentration was measured using a Qubit 2.0 Fluorometer (Invitrogen, Thermo Fisher Scientific, Waltham, MA, USA), while RNA purity was assessed based on the A260/A280 ratio using a NanoDrop 2000 spectrophotometer (Thermo Fisher Scientific, USA). Samples with an A260/A280 ratio between 1.8 and 2.0 were used for subsequent analyses.

### 2.5. cDNA Synthesis and RT-qPCR

Total RNA was reverse-transcribed using a miRNA-specific reverse transcription kit. Quantitative real-time PCR (RT-qPCR) (Applied Biosystems, Singapore) was performed for ten target miRNAs (let-7c, miR-10b, miR-27a, miR-29b, miR-31, miR-34a, miR-142, miR-143, miR-17-5p, and miR-200c). U6 small nuclear RNA (U6 snRNA) and SNORD48 were used as endogenous reference controls for normalization. These reference genes were selected because they are widely used as endogenous controls in miRNA RT-qPCR studies and have been demonstrated to exhibit stable expression and provide reliable normalization in formalin-fixed, paraffin-embedded (FFPE) tissue samples [33,34]. All qPCR reactions were performed with at least two technical replicates. Amplification performance was assessed by evaluating amplification curves and the reproducibility of Ct values across technical replicates. Relative miRNA expression levels were calculated using the 2^−ΔΔCt^ method. For statistical comparisons, the underlying ΔCt values were used because these values preserve the appropriate scale of the qPCR measurements and avoid statistical analysis of derived fold-change estimates. The 2^−ΔΔCt^ values were used for descriptive presentation of relative expression. For graphical and tabular presentation, downregulated miRNAs were additionally expressed using a fold-regulation convention, in which values below 1 were represented as the negative reciprocal of the corresponding fold change. Therefore, negative values shown in the descriptive tables indicate downregulation and do not represent negative 2^−ΔΔCt^ values. For each target miRNA, Ct values were normalized to the mean Ct value of U6 and SNORD48. The mean ΔCt value obtained from all 21 adjacent non-tumor pancreatic tissue samples was used as the reference for the tumor samples. Thus, tumor miRNA expression was evaluated relative to the overall mean expression level of the 21 non-tumor tissue samples rather than by a patient-specific tumor-to-matched-normal comparison.

### 2.6. Statistical Analysis

Continuous variables were summarized as mean ± standard deviation or median (interquartile range), depending on distribution; categorical variables were summarized as counts and percentages. Comparisons of miRNA expression between tumor and adjacent non-tumor tissues were performed using Student’s *t*-test or the Mann–Whitney U test, as appropriate. Group comparisons used the chi-square test, Student’s *t*-test, one-way ANOVA, or the Mann–Whitney U test, as appropriate. For prognostic analyses, miRNA expression values were dichotomized at the median into low and high categories. The clinicopathological variables initially evaluated in the univariable analyses included age, sex, histological subtype, perineural invasion, and the dichotomized expression levels of the ten investigated miRNAs. To reduce the risk of model overfitting in this relatively small cohort, only variables with an univariable *p* value < 0.05 were entered into the multivariable logistic regression and Cox proportional hazards models. Local recurrence was analyzed as a binary outcome, categorized as present or absent during the available follow-up period. Binary logistic regression was used to evaluate factors associated with the occurrence of local recurrence. This analysis was intended to assess the occurrence of local recurrence during the follow-up period and did not incorporate time to recurrence, censoring, or competing events such as death or distant metastasis. Consequently, the final multivariable models included only variables that met this predefined selection criterion. Variables associated with DFS or OS at *p* < 0.05 in the univariate analyses were entered into multivariable Cox proportional-hazards models. Results from the regression models are reported as odds ratios (ORs) or hazard ratios (HRs) with 95% confidence intervals (CIs). All analyses were performed with IBM SPSS Statistics for Windows, version 21.0 (IBM Corp., Armonk, NY, USA). A two-sided *p* value < 0.05 was considered statistically significant. Because this study was designed as an exploratory, hypothesis-generating analysis, no formal adjustment for multiple comparisons (such as Bonferroni correction or false discovery rate control) was applied. Therefore, the reported *p* values should be interpreted as exploratory and require confirmation in independent validation cohorts.

## 3. Results

### 3.1. Patient Characteristics

Fifty patients with resected PDAC who received adjuvant chemotherapy were included (Table 1). The mean age at diagnosis was 61.6 ± 11.2 years, and 34 patients (68%) were male. Adjacent non-tumor pancreatic tissue was available for paired analysis in 21 of the 50 patients. Median DFS was 8 months (95% CI 5.4–10.6), whereas median OS was 16 months (95% CI 11.4–20.6).

### 3.2. miRNA Expression in Tumor Versus Adjacent Non-Tumor Tissue

**Figure 1:**
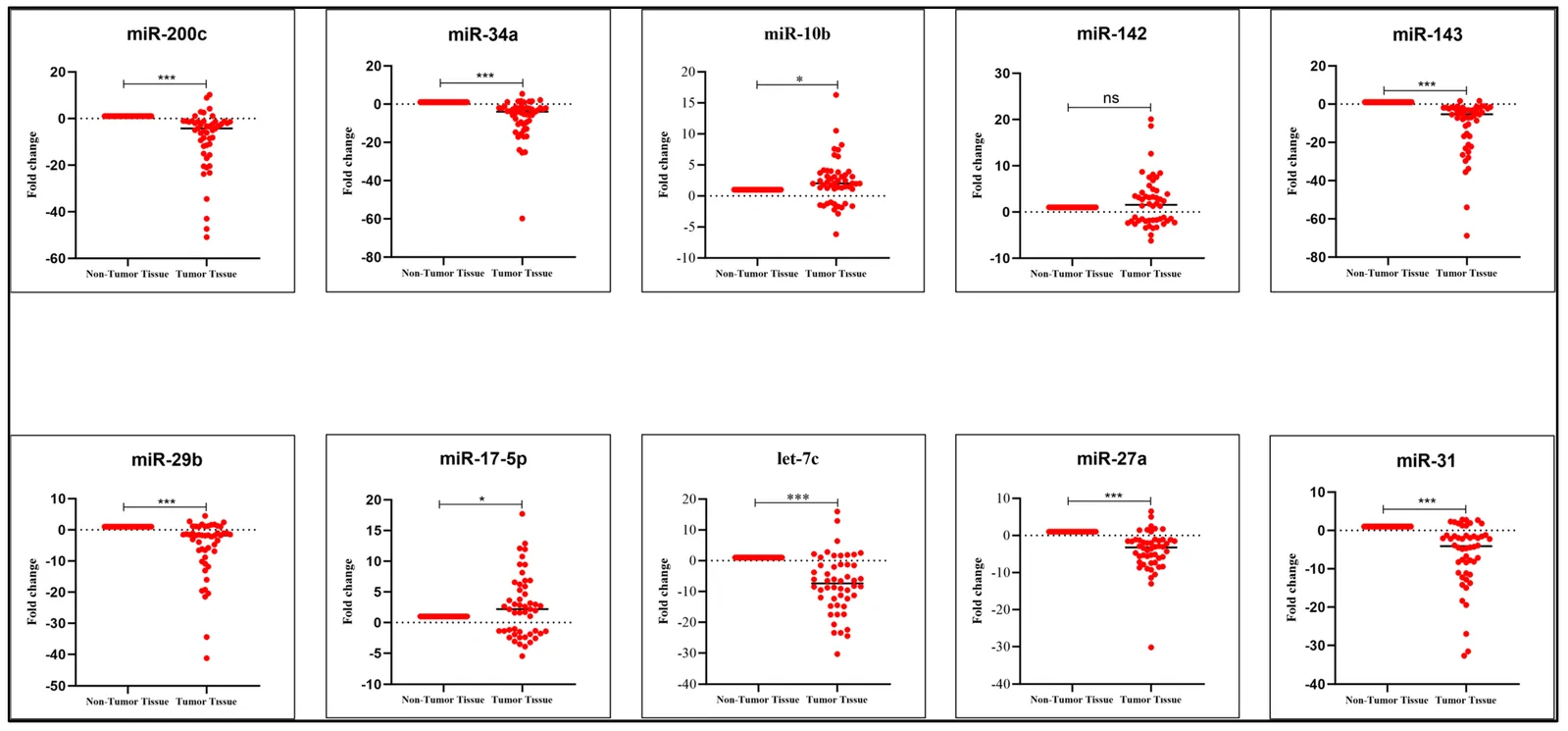
Distribution of miRNA expression in tumor and adjacent non-tumor pancreatic tissues. Individual miRNA expression values are shown as scatter plots for the ten investigated miRNAs. Tumor tissue values are presented relative to the mean expression level of the 21 adjacent non-tumor tissue samples, which was used as the reference. Each dot represents an individual tumor sample. The dashed horizontal line represents the reference level of the adjacent non-tumor tissue samples. Statistical significance was determined using the appropriate comparison test. * *p* < 0.05, *** *p* < 0.001; ns, not significant.

In the 21 paired samples, seven of the ten miRNAs were differentially expressed between tumor and adjacent non-tumor tissue. Let-7c, miR-27a, miR-29b, miR-31, miR-34a, miR-143, and miR-200c were significantly downregulated in tumor tissue, whereas miR-10b was upregulated (all *p* < 0.05). No significant differences were observed for miR-142 or miR-17-5p (Figure 1).

### 3.3. Association with Local Recurrence

For prognostic analyses, all 50 patients were considered, and miRNA expression was dichotomized at the median into low and high categories. When patients with and without local recurrence were compared, miR-143 was the only miRNA showing a significant difference, with higher fold-change values in the recurrence group (Table 2). Among clinicopathological variables, perineural invasion also discriminated between the two groups. In multivariable binary logistic regression including variables with *p* < 0.05 in the univariate analysis, two factors emerged as independent correlates of local recurrence (Table 3). High miR-143 expression was associated with an approximately fourfold increase in the odds of local recurrence (OR 4.14, 95% CI 1.09–15.67, *p* = 0.036). Perineural invasion was retained as the second independent variable (*p* = 0.025); the corresponding effect estimate is reported in Table 3, with the reference category indicated.

### 3.4. Association with Distant Metastasis

No miRNA differed significantly between patients with and without distant metastasis at any anatomical site, and no clinicopathological variable reached statistical significance in this analysis (Table 4). Trends toward higher miR-10b and lower miR-200c in the metastatic group did not reach the prespecified threshold and are not discussed further.

### 3.5. Disease-Free and Overall Survival

Univariate Kaplan–Meier analysis identified three variables associated with survival outcomes. Adenosquamous histology was associated with both shorter DFS and shorter OS. High miR-29b expression was associated with shorter DFS, and high miR-142 expression was associated with shorter OS (Table 5). In multivariable Cox regression for DFS, including variables with *p* < 0.05 at the univariate level, adenosquamous histology and miR-29b expression remained significantly associated with. Adenosquamous histology was associated with a hazard ratio of 4.11 for DFS (95% CI 1.61–10.76, *p* = 0.003), and high miR-29b expression with a hazard ratio of 1.99 (95% CI 1.08–3.66, *p* = 0.027). For OS, only adenosquamous histology retained independent prognostic value (HR 5.10, 95% CI 1.82–14.30); miR-142 did not remain significant after adjustment.

The principal prognostic findings of the multivariable analyses are summarized in Table 6. Among the ten investigated miRNAs, only high miR-143 expression remained independently associated with local recurrence, while high miR-29b expression remained independently associated with shorter disease-free survival.

## 4. Discussion

In a single-center cohort of 50 patients with resected PDAC who received adjuvant chemotherapy, three findings emerged. First, seven of ten miRNAs were downregulated and one was upregulated in tumor tissue compared with paired adjacent non-tumor pancreas. The observed pattern was consistent with the predominantly tumor-suppressive function of the seven downregulated miRNAs and with the oncogenic function previously attributed to miR-10b in cancer growth and metastasis [24]. Second, residual tumor expression of miR-143 remained significantly associated with local recurrence in the multivariable analysis, and residual tumor expression of miR-29b remained significantly associated with disease-free survival. Third, adenosquamous histology was the only variable independently associated with both disease-free and overall survival. The most informative aspect of these data is the apparent paradox surrounding miR-143 [29] and miR-29b [28], both of which behave as tumor suppressors in the tumor-versus-normal comparison and yet identify, within the tumor compartment, the patients with the worst clinical trajectory.

The observed paradox is best understood by recognizing that the two analyses ask different biological questions. In this regard, the paired comparison between tumor and adjacent non-tumor tissue measures the average loss of miRNA expression that accompanies neoplastic transformation. The prognostic analysis, in contrast, ranks tumors against each other and asks whether residual variability above or below the cohort median carries clinical information. An miRNA that drops in absolute terms during transformation can therefore still provide useful prognostic separation if its residual abundance reflects the activity of a biological program that drives recurrence in the resected and chemotherapy-treated setting. Several biological mechanisms, including stromal remodeling, extracellular matrix regulation, and epithelial-to-mesenchymal transition, have been proposed in previous studies to explain the biological functions of miR-143 and miR-29b [35,36]. However, these mechanisms were not directly investigated in the present study and therefore should be considered as possible biological explanations rather than conclusions supported by our data.

miR-143 and miR-29b are highly expressed in normal stromal cells and act on targets that regulate collagen deposition, fibroblast activation, and matrix stiffness [37]. Kwon and colleagues showed that miR-29 family members are abundant in the pancreatic stroma and modulate the cross-talk between cancer-associated fibroblasts and tumor cells, with effects on tumor growth and chemoresistance [38]. In bulk tumor tissue, the measured signal therefore reflects a mixture of epithelial and stromal contributions, and a relatively high residual signal in resected PDAC may indicate a more abundant or more active desmoplastic compartment. Desmoplasia in PDAC is associated with elevated interstitial pressure, impaired drug delivery, and a microenvironment that supports the survival of residual disease after surgery [39]. One possible explanation is that higher residual miR-29b expression may reflect differences in stromal composition or activity. However, because bulk FFPE tissue was analyzed without compartment-specific evaluation, the present study cannot determine whether the observed prognostic associations originate from tumor epithelial cells, stromal cells, or both. A similar interpretation can be applied to miR-143, whose stromal expression contributes to fibroblast activation and matrix remodeling [40], two processes that are linked to local invasion and to the niches that sustain microscopic residual disease in the resection bed [41].

Another possible biological explanation may involve EMT-related pathways that have previously been associated with miR-143 and miR-29 family members. Nevertheless, since EMT-associated markers or signaling pathways were not evaluated in this study, this interpretation remains speculative. Both miR-143 [42] and the miR-29 family [43] modulate EMT-related programs, including cadherin switching, vimentin expression, and the activity of transcription factors such as SNAI1 and ZEB1 [44]. EMT is one of the routes by which PDAC cells survive cytotoxic stress and seed local recurrence [45], and the relationship between EMT regulators and clinical outcome is known to be non-monotonic, since both excess and insufficient activity of these regulators can produce phenotypes that favor dissemination [46]. The association between higher residual miR-143 and local recurrence, and between higher residual miR-29b and shorter DFS, fits this non-monotonic pattern, in which the prognostic signal lies in the deviation from a tightly regulated baseline rather than in the simple direction of change.

The behavior of miR-29b in the literature is consistent with this context-dependent reading. Some series have reported that low miR-29b correlates with shorter survival, including reports in breast cancer [47] and glioblastoma [48]. Conversely, other studies, particularly those that examined miR-29 family members in PDAC stroma, have found that higher levels accompany aggressive disease [38]. The discrepancy is therefore reduced once the tissue compartment, the disease subtype, and the treatment context are considered separately. In the present cohort, in which all patients received adjuvant chemotherapy after resection, the prognostic signal of miR-29b probably reflects stromal activity in the residual tumor bed rather than a tumor-cell-autonomous tumor-suppressive function. The same caveat applies to miR-143, which has been described as a tumor suppressor in non-small cell lung cancer [49] but whose role in PDAC stroma and in resected tumor tissue has been examined less systematically. Nevertheless, the available evidence regarding the stromal roles of miR-143 and miR-29 family members in PDAC remains context-dependent and, in some instances, inconsistent across studies. Therefore, the proposed stromal interpretation should be considered as one of several plausible biological explanations rather than a definitive mechanistic conclusion.

In our study, adenosquamous histology emerged as the strongest clinicopathological predictor in this cohort and was independently associated with both DFS and OS. Adenosquamous carcinoma of the pancreas is a recognized variant with biology that differs from conventional PDAC, including a higher proliferation index, a distinct mutational background, and patterns of squamous differentiation that have been linked to worse outcomes after resection [50]. The independent prognostic role observed here adds to the existing evidence that this subtype warrants distinct consideration in postoperative risk stratification.

The findings of the present study should be interpreted in the light of several limitations. The sample size is small and the cohort is derived from a single center, which limits statistical power and external validity, as also reflected by the wide confidence intervals around the point estimates of the regression models. The relatively small sample size may also have affected the stability of the multivariable logistic and Cox regression models. Because only 50 patients were included and the number of outcome events was limited for some endpoints, the events-per-variable ratio was modest. Consequently, the reported odds ratios and hazard ratios should be interpreted with caution, as the relatively wide confidence intervals may reflect statistical uncertainty rather than precise effect estimates. Although only a limited number of variables were included in each final multivariable model, larger prospective cohorts will be required to confirm the robustness of these associations. In addition, paired adjacent non-tumor tissue was available for only 21 patients. Therefore, comparisons between tumor and normal tissues were based on a relatively small paired subset and may not fully represent the biological variability of the entire study population. Another important limitation is the lack of an independent validation cohort. Consequently, biomarker discovery and evaluation were performed within the same patient population, and the reproducibility of these findings remains to be established. In addition, multiple miRNAs were evaluated across several clinical endpoints without formal adjustment for multiple comparisons. Consequently, the possibility of type I error and false-positive findings cannot be excluded, and the reported associations should be considered exploratory until confirmed in larger independent validation cohorts. Future studies should incorporate approaches that allow cell-type-specific assessment of miRNA expression within the PDAC tumor microenvironment. Laser-capture microdissection could be used to separately isolate malignant epithelial/tumor and stromal compartments from FFPE sections before miRNA quantification, thereby reducing the confounding effect of differences in tumor purity and stromal abundance. Complementary approaches, including immunohistochemistry or multiplex immunofluorescence with cell-type-specific markers, could further characterize the spatial distribution of malignant epithelial cells and fibroblastic stromal populations. More advanced spatial and single-cell-based approaches may additionally help resolve cell-specific molecular programs and interactions within the tumor microenvironment. Such studies would be important to determine whether the prognostic associations observed for miR-143 and miR-29b primarily reflect expression within malignant tumor cells, fibroblasts, other stromal populations, or a combination of these compartments. The retrospective design introduces the risk of selection and information bias, and the dichotomization of miRNA expression at the median, although standard, can amplify noise in small cohorts. Functional validation of the proposed mechanisms was not performed, and the inferences regarding stromal activity and EMT remain hypothesis-generating. Finally, the analysis was restricted to tumor tissue, and parallel measurements in plasma or in laser-microdissected stromal compartments would help separate the epithelial and stromal contributions to the prognostic signal. If validated in larger prospective cohorts, tissue expression of miR-143 and miR-29b may complement established clinicopathological prognostic factors, such as tumor stage, nodal status, resection margin status, and histological subtype, to improve postoperative risk stratification in patients with resected PDAC. Such biomarkers could potentially help identify patients at increased risk of early recurrence who may benefit from intensified postoperative surveillance, enrollment in clinical trials, or future biomarker-guided therapeutic strategies. Nevertheless, the present findings should be regarded as preliminary, and these miRNAs are not yet suitable for routine clinical use until their prognostic value has been independently validated and standardized across larger patient populations.

## 5. Conclusions

In patients with resected PDAC who received adjuvant chemotherapy, higher residual tumor expression of miR-143 and miR-29b was independently associated with local recurrence and shorter disease-free survival, respectively. These findings are exploratory and require validation in larger independent prospective cohorts before these biomarkers can be considered for clinical risk stratification.

## Figures and Tables

**Table 1:** Demographic, clinical, pathological, and treatment characteristics of the study group (n = 50).

Characteristic	Value
**Age at diagnosis, years (mean ± SD)**	61.6 ± 11.2
**Sex, n (%)**	
Male	34 (68.0)
Female	16 (32.0)
**Smoking status, n (%)**	
No	36 (72.0)
Yes	14 (28.0)
**Alcohol use, n (%)**	
No	45 (90.0)
Yes	5 (10.0)
**ECOG performance status at diagnosis, n (%)**	
0	8 (16.0)
1	37 (74.0)
2	4 (8.0)
3	1 (2.0)
**CA19-9 level at diagnosis, n (%)**	
Normal	14 (28.0)
High	36 (72.0)
**Histological subtype, n (%)**	
Adenocarcinoma	44 (88.0)
Adenosquamous carcinoma	6 (12.0)
**Local invasion, n (%)**	
Negative	4 (8.0)
Positive	46 (92.0)
**Vascular invasion, n (%)**	
Negative	41 (82.0)
Positive	9 (18.0)
**Perineural invasion, n (%)**	
Negative	8 (16.0)
Positive	42 (84.0)
**Surgical technique, n (%)**	
Whipple procedure	45 (90.0)
Total/subtotal pancreatectomy	5 (10.0)
**Surgical margin, n (%)**	
Negative	43 (86.0)
Positive	7 (14.0)
**TNM stage, n (%)**	
Stage I	4 (8.0)
Stage II	35 (70.0)
Stage III	11 (22.0)
**Treatment, n (%)**	
Chemotherapy	30 (60.0)
Chemoradiotherapy	20 (40.0)
**Treatment regimen, n (%)**	
Gemcitabine/gemcitabine + capecitabine	32 (64.0)
Gemcitabine + cisplatin	3 (6.0)
Gemcitabine + oxaliplatin	7 (14.0)
Gemcitabine + nab-paclitaxel	2 (4.0)
FOLFIRINOX	5 (10.0)
FOLFOX	1 (2.0)
**Local recurrence, n (%)**	
No	31 (62.0)
Yes	19 (38.0)
**Distant metastasis, n (%)**	
No	6 (12.0)
Yes	44 (88.0)
**Survival duration, n (%)**	
<12 months	18 (36.0)
12–24 months	18 (36.0)
>24 months	14 (28.0)

SD, standard deviation. ECOG: Eastern Cooperative Oncology Group performance status.

**Table 2:** miRNA expression (fold-change relative to adjacent non-tumor tissue) in patients with versus without local recurrence.

miRNA	Recurrence (+), Mean	Recurrence (−), Mean	*p* Value
let-7c	−7.082	−7.867	0.779
miR-10b	2.215	2.352	0.773
miR-27a	−2.771	−4.998	0.387
miR-29b	−4.099	−6.399	0.773
miR-31	−3.804	−7.906	0.387
miR-34a	−5.188	−8.575	0.387
miR-142	1.779	2.215	0.773
miR-143	−8.266	−12.874	0.043
miR-17-5p	2.712	2.548	0.773
miR-200c	−6.820	−9.970	0.773

Relative expression was calculated using the 2^−ΔΔCt^ method. For statistical analyses, comparisons were performed using ΔCt values. For descriptive presentation only, relative expression values were expressed as fold regulation: values > 1 indicate upregulation, whereas values < 1 were expressed as the negative reciprocal to indicate downregulation. Thus, negative values in this table are presentation values and do not represent negative 2^−ΔΔCt^ values.

**Table 3:** Multivariable binary logistic regression for local recurrence.

Variable	β	*p* Value	OR (95% CI)
Perineural invasion (positive vs. negative)	−2.126	0.025	0.12 (0.019–0.76)
miR-143 expression (high vs. low)	1.421	0.036	4.14 (1.09–15.67)

Variables with *p* < 0.05 in the univariable analysis were entered into the multivariable logistic regression model. Perineural invasion was coded as positive versus negative, with the negative group used as the reference category. Odds ratios (ORs) and 95% confidence intervals (CIs) are reported.

**Table 4:** miRNA expression (fold-change relative to adjacent non-tumor tissue) in patients with versus without distant metastasis.

miRNA	Metastasis (+), Mean	Metastasis (−), Mean	*p* Value
let-7c	−7.457	−8.387	0.824
miR-10b	2.484	0.951	0.269
miR-27a	−4.176	−3.970	0.811
miR-29b	−5.278	−7.335	0.210
miR-31	−6.479	−5.383	0.811
miR-34a	−7.116	−8.547	0.612
miR-142	1.899	3.154	0.420
miR-143	−11.392	−9.147	0.282
miR-17-5p	2.645	2.355	0.905
miR-200c	−9.584	−2.828	0.199

Relative expression was calculated using the 2^−ΔΔCt^ method. For statistical analyses, comparisons were performed using ΔCt values. For descriptive presentation only, relative expression values were expressed as fold regulation: values > 1 indicate upregulation, whereas values < 1 were expressed as the negative reciprocal to indicate downregulation. Thus, negative values in this table are presentation values and do not represent negative 2^−ΔΔCt^ values.

**Table 5:** Median disease-free survival and overall survival according to miRNA expression.

Variable	Category	Median DFS (Months)	*p* (log-Rank)	Median OS (Months)	*p* (log-Rank)
let-7c	Low expression	10.0	0.555	18.0	0.318
	High expression	8.0		14.0	
miR-10b	Low expression	10.0	0.445	17.0	0.537
	High expression	7.0		11.0	
miR-27a	Low expression	9.0	0.813	19.0	0.250
	High expression	8.0		14.0	
miR-29b	Low expression	10.0	0.045	18.0	0.074
	High expression	7.0		14.0	
miR-31	Low expression	11.0	0.123	19.0	0.121
	High expression	7.0		14.0	
miR-34a	Low expression	9.0	0.354	19.0	0.329
	High expression	8.0		13.0	
miR-142	Low expression	8.0	0.326	19.0	0.038
	High expression	9.0		14.0	
miR-143	Low expression	11.0	0.098	19.0	0.269
	High expression	6.0		10.0	
miR-17-5p	Low expression	7.0	0.905	14.0	0.726
	High expression	10.0		18.0	
miR-200c	Low expression	9.0	0.814	15.0	0.878
	High expression	8.0		18.0	

DFS, disease-free survival; OS, overall survival. miRNA expression dichotomized at the median. Bold indicates *p* < 0.05.

**Table 6:** Summary of the prognostic significance of miR-143 and miR-29b in resected PDAC.

Biomarker	Clinical Endpoint	Multivariable Analysis	Effect Estimate	*p* Value
miR-143 (high expression)	Local recurrence	Binary logistic regression	OR = 4.14 (95% CI: 1.09–15.67)	0.036
miR-29b (high expression)	Disease-free survival	Cox proportional-hazards model	HR = 1.99 (95% CI: 1.08–3.66)	0.027

OR, odds ratio; HR, hazard ratio; CI, confidence interval. Only statistically significant independent prognostic associations are presented.

## Data Availability

The data presented in this study are available on request from the corresponding author.
